# Kv7 channel activation reduces brain endothelial cell permeability and prevents kainic acid-induced blood-brain barrier damage

**DOI:** 10.1152/ajpcell.00709.2023

**Published:** 2024-01-29

**Authors:** Camilla Celentano, Lidia Carotenuto, Francesco Miceli, Giusy Carleo, Brunella Corrado, Giulia Baroli, Stefania Iervolino, Raffaele Vecchione, Maurizio Taglialatela, Vincenzo Barrese

**Affiliations:** ^1^Department of Neuroscience, Reproductive Sciences and Dentistry, University of Naples Federico II, Naples, Italy; ^2^Interdisciplinary Research Centre on Biomaterials, University of Naples Federico II, Naples, Italy; ^3^Center for Advanced Biomaterials for Health Care, Istituto Italiano di Tecnologia, Naples, Italy

**Keywords:** blood-brain barrier, brain endothelial cells, epilepsy, Kv7 channels, retigabine

## Abstract

Ion channels in the blood-brain barrier (BBB) play a main role in controlling the interstitial fluid composition and cerebral blood flow, and their dysfunction contributes to the disruption of the BBB occurring in many neurological diseases such as epilepsy. In this study, using morphological and functional approaches, we evaluated the expression and role in the BBB of Kv7 channels, a family of voltage-gated potassium channels including five members (Kv7.1-5) that play a major role in the regulation of cell excitability and transmembrane flux of potassium ions. Immunofluorescence experiments showed that Kv7.1, Kv7.4, and Kv7.5 were expressed in rat brain microvessels (BMVs), as well as brain primary- and clonal (BEND-3) endothelial cells (ECs). Kv7.5 localized at the cell-to-cell junction sites, whereas Kv7.4 was also found in pericytes. The Kv7 activator retigabine increased transendothelial electrical resistance (TEER) in both primary ECs and BEND-3 cells; moreover, retigabine reduced paracellular dextran flux in BEND-3 cells. These effects were prevented by the selective Kv7 blocker XE-991. Exposure to retigabine also hyperpolarized cell membrane and increased tight junctions (TJs) integrity in BEND-3 cells. BMVs from rats treated with kainic acid (KA) showed a disruption of TJs and a selective reduction of Kv7.5 expression. In BEND-3 cells, retigabine prevented the increase of cell permeability and the reduction of TJs integrity induced by KA. Overall, these findings demonstrate that Kv7 channels are expressed in the BBB, where they modulate barrier properties both in physiological and pathological conditions.

**NEW & NOTEWORTHY** This study describes for the first time the expression and the functional role of Kv7 potassium channels in the blood-brain barrier. We show that the opening of Kv7 channels reduces endothelial cell permeability both in physiological and pathological conditions via the hyperpolarization of cell membrane and the sealing of tight junctions. Therefore, activation of endothelial Kv7 channels might be a useful strategy to treat epilepsy and other neurological disorders characterized by blood-brain barrier dysfunction.

## Graphical abstract

**Figure:**
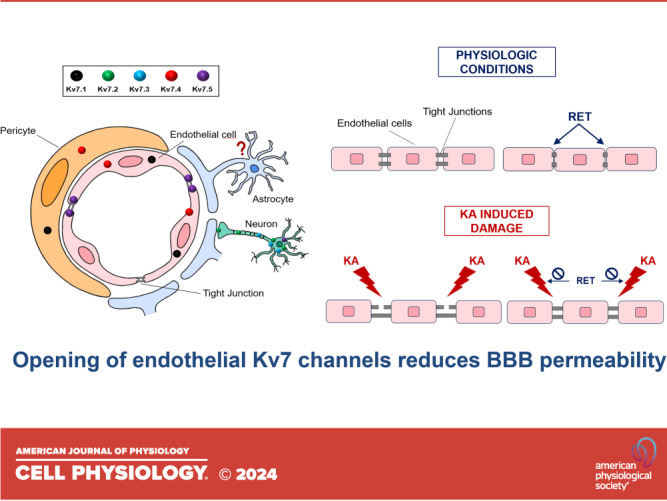

## INTRODUCTION

The blood-brain barrier (BBB) regulates the movement of toxins, pathogens, inflammatory mediators, cells, solutes, and ions between the blood and the brain parenchyma, thus playing a crucial role in the modulation of neuronal function within the central nervous system (CNS; 1). In contrast to most “peripheral” small vessels, the continuous nonfenestrated capillaries in the CNS are constituted by endothelial cells (ECs) connected by tight junctions (TJs) formed by adhesion and adaptor proteins such as occludin, claudin-5, and zonula occludens. In addition, astrocytes and pericytes (PCs), which form along with ECs in the neurovascular unit, contribute to the development and maintenance of barrier properties (2). Alteration of the BBB is a common finding in several neurological disorders, including epilepsy. In particular, BBB widening is known to promote the onset of seizures via multiple mechanisms including the alteration of extracellular potassium concentration, a sudden increase in the levels of excitatory neurotransmitters such as glutamate, and enhancement of neuroinflammation (3). Conversely, prolonged epileptic activity increases the production of reactive oxygen species (ROS), enhances the activity of matrix metalloproteases that degrade TJs and augments neuroinflammation, thus causing BBB dysfunction (4). Therefore, preventing BBB alterations might represent a useful strategy to treat epilepsy.

Given the reduced permeability of brain capillaries, control of interstitial fluid ionic composition is strictly dependent on specific transport proteins located at the BBB. To date, along with ion transporters such as sodium-potassium-chloride cotransporter (5) and sodium-hydrogen exchanger (6), few classes of ion channels have been identified in brain ECs and PCs. Among them, the expression of sodium (Na^+^) (7), calcium (Ca^2+^) (8), potassium (K^+^) (9–13), and nonselective cation channels (14) has been reported in both primary and clonal brain microvascular ECs. These studies also highlighted that brain endothelial ion channels regulate multiple processes including neuronal activity and cerebral blood flow. Moreover, functional alterations of endothelial K^+^ channels have been found in neurological diseases such as brain ischemia and multiple sclerosis, where they contribute to the disruption of the BBB and the exacerbation of the diseases (15, 16). To date, the role of endothelial K^+^ channels in epilepsy has been poorly investigated.

Among the heterogeneous family of K^+^ channels, voltage-gated Kv7 channels (Kv7.1–5) show peculiar tissue distribution and pathophysiological role (17). Kv7.2, Kv7.3, and Kv7.5 participate in the regulation of neuronal excitability, and mutations in these genes are associated with forms of epilepsy showing different degrees of severity (18). Instead, Kv7.1, Kv7.4, and Kv7.5 are found in vascular smooth muscle cells, where they regulate arterial tone (19). Kv7.1 is also expressed in nonexcitable cells such as epithelial cells where it regulates electrolyte balance, provides the driving force for chloride flux (17, 20), and contributes to myoinositol and iodide transport (21, 22). More recently, endothelial Kv7.4 and Kv7.5 (and possibly Kv7.1) have been found to regulate ECs-mediated vasorelaxation in pig coronary artery (23) and rat mesenteric artery (24).

In the present work, we adopted a combined approach using morphological and functional evaluations to investigate the expression and function of Kv7 channels in brain ECs and assess their involvement in the alteration of the BBB induced by kainic acid in different in vitro and in vivo experimental models.

## MATERIALS AND METHODS

### Animals

Three week-old male Sprague-Dawley rats (Charles River Laboratories, Italy) were housed in groups of two per cage under controlled conditions (temperature: 21 ± 1°C, relative humidity: 60 ± 10%, and 12/12 h light cycle). Food and water were available ad libitum. The experiments were approved by the Italian Ministry of Health (No. 1130/2020-PR) and performed in agreement with the ARRIVE (Animals in Research: Reporting In Vivo Experiments) guidelines, with the guidelines released by the Italian Ministry of Health (D.L. 26/14) and the European Union Directive 2010/63/EU.

### Kainic Acid Status Epilepticus Model

Kainic acid (KA) dissolved in saline solutions (0.9 g/L NaCl) was administered intraperitoneally (ip) to rats until the onset of status epilepticus (SE) as previously described (25, 26), with some changes. Rats received a first injection of KA (15 mg/kg); then, they were returned to their cage and monitored for 30 min. Visual assessment of seizure severity was performed using the modified Racine scale (25); if severe seizures (defined as ≥ stage 3, according to the modified Racine scale) did not occur, rats received up to two more injections of lower dose of KA (5 mg/kg). SE was defined as the occurrence of at least 10 severe seizures in 1 h; rats that did not develop SE after the third KA injection were considered as nonresponders and excluded from the analysis. SE was terminated with 5 mg/kg midazolam (ip). Sham-treated rats received intraperitoneal injections of saline. Rats were culled 24 h after the procedure, brains were dissected and used for Western blot experiments and BMVs isolation.

### Isolation of BMVs

BMVs were isolated according to Brzica et al. (27). Briefly, whole brains or selected areas were homogenated in brain microvessel buffer (BMB), containing (in mM): 300 mannitol, 5 EGTA, and 12 Tris-base, pH 7.4. BMB containing 26% dextran (wt/vol) was added to the homogenate, to get a final concentration of 16% dextran (wt/vol). The suspension was then centrifuged at 5,000 *g* for 15 min at 4°C. Pellets were resuspended with BMB-dextran (16%); centrifugation in BMB-dextran was repeated 2–3 more times. The obtained microvessels were resuspended in BMB, plated on 12 mm diameter glass coverslips, and let adhere for 1 h at 37°C.

### Isolation of Rat Brain ECs

Primary brain ECs were isolated from rats according to Assmann et al. (28). Brains were dissected and homogenated in Dulbecco’s modified eagle medium (DMEM)-F12 containing 1% l-glutamine and 1% penicillin/streptomycin, and centrifuged at 1,350 *g* for 5 min at 4°C. Pellets were resuspended in 18% dextran solution and spun at 6,080 *g* for 10 min at 4°C. The obtained BMV fraction was digested in DMEM containing 1 mg/mL collagenase/dispase, 4 µg/mL DNase I, and 0.146 µg/mL Nα-Tosyl-l-lysine chloromethyl ketone hydrochloride (TLCK) for 1 h at 37°C. The suspension was centrifuged at 1,350 *g* for 5 min at 22°C–24°C (room temperature, RT). The resulting pellet was washed with PBS, resuspended in full medium [DMEM-F12 with 1% l-glutamine, 1% antibiotic/antimycotic, 20% fetal bovine serum (FBS), 30 µg/mL endothelial cells growth factor, 15 U/mL heparin, and 8 µg/mL puromycin] and seeded onto collagen IV-coated plates or coverslips (passage 0). After 3 days, primary ECs were grown in full medium without puromycin. Experiments were performed on p0 and p1 primary ECs. For transendothelial electrical resistance (TEER) experiments, primary ECs (p1) were seeded onto collagen IV-coated Transwell inserts (pore size 0.4 µm, membrane area 0.33 cm^2^; Corning Inc, Corning). Inserts were put in a 24-well plate in which C6 cells were seeded at the bottom of the well. Primary ECs were maintained in full medium up to 96 h.

### Isolation of Rat Brain PCs

Primary brain PCs were isolated from rats as previously described (29) with slight modifications. BMVs fractions were obtained as described in the previous section (Isolation of brain ECs) and digested with DMEM containing 1.05 mg/mL type II collagenase and 58.5 U/mL DNAse I for 10 min at RT. The suspension was centrifuged at 800 *g* for 8 min at 4°C, resuspended in growing medium (low glucose DMEM with 1% penicillin/streptomycin, 20% FBS, 42 µg/mL Insulin, 38 µg/mL Transferrin, 50 ng/mL Sodium Selenite, 100 µg/mL heparin, and Smooth Muscle Growth Supplement), and seeded in T-25 flasks (passage 0). For immunofluorescence experiments, after reaching confluence, primary PCs were plated onto 12 mm glass coverslips. Experiments were performed on p0-p2 cells.

### Cell Culture

BEND-3, C6, and CHO cells were maintained in DMEM supplemented with 10% FBS, 1% l-glutamine, and 1% penicillin/streptomycin (growing medium) in a humidified atmosphere at 37°C with 5% CO_2_ and passed every 3 days. For coculture experiments (dextran flux, TEER, and immunofluorescence), BEND-3 cells were seeded in Transwell inserts at a density of 2,000 cells/well. Inserts were placed in a 24-well plate in which C6 cells were seeded at the bottom of the well. After 4 days, growing medium was switched to DMEM supplemented with 2% FBS, 1% l-glutamine, and 1% penicillin/streptomycin (low-serum medium); cells were maintained in this medium up to 96 h. For some sets of experiments (qPCR and Western blot depicted in Fig. 3*A* and Supplemental Fig. S4; electrophysiological experiments), to mimic the exposure of ECs to factors released by astrocytes, BEND-3 cells were grown in monoculture and incubated up to 96 h with C6 cells-conditioned medium diluted 1:1 with DMEM (CM).

### Transfection

CHO cells plated on 12 mm coverslips were transfected with plasmids encoding for Kv7 subunits using Lipofectamine 2000 (Thermo Fisher, Monza, Italy), according to the manufacturer’s instructions. CHO cells transfected with an empty plasmid vector (pcDNA3.1) were used as controls for immunofluorescence experiments.

### Immunofluorescence, Confocal Imaging, and Images Analysis

Cells or BMVs plated on coverslips or Transwell inserts were fixed with 4% paraformaldehyde, incubated with 0.1 M glycine, permeabilized/blocked with 1% BSA, 0.1% Triton X-100 diluted in PBS (blocking buffer) for 1 h at RT, and incubated with primary antibodies overnight at 4°C. Coverslips and Transwell inserts were then washed with PBS and incubated for 1 h with secondary antibodies conjugated to Alexa Fluor 488 or Alexa Fluor 555 (see Supplemental Table S1 for primary and secondary antibodies). Samples were imaged with a Zeiss LSM 700 confocal microscope (Carl Zeiss, Jena, Germany) with a ×63 objective. Images were analyzed using Fiji software (https://imagej.net/software/fiji/). Tight junction organization rate (TiJOR) was measured using images with zonula occludens-1 (ZO-1) staining of BEND-3 cells grown on Transwell inserts, as previously described (30), with slight changes. Briefly, a grid formed by 23 lines was randomly applied to the images, and a multiple-line scan analysis of the fluorescence intensity of ZO-1 was performed. “Hits” were identified as peaks of fluorescence crossing a line of the grid; peaks were defined as values of fluorescent intensity above an arbitrary threshold (10% of the maximal fluorescent intensity of the image). The ratio between the number of hits along a line and the length of the same line was calculated; TiJOR values for each image were calculated as the average of the ratios of the 23 lines of the grid. To quantify the expression of TJs markers and Kv7 channels in BMVs, the length of fluorescence of ZO-1, Kv7.1, Kv7.4, and Kv7.5 was measured and calculated relative to the total length of the vessel, measured using bright-field images, as previously described (31).

### TEER Measurements

TEER was measured at RT using a Millicell-ERS ohmmeter (Millipore-Merck Life Science, Milan, Italy) with electrodes inserted on both sides of the Transwell insert. The alternating current applied between the electrodes was within ± 10 µA at a frequency of 12.5 Hz. TEER values expressed in Ω × cm^2^ were calculated by subtracting the resistance of a blank insert (background) from the resistance of the BEND-3 and primary ECs monolayer and multiplying this value by the effective surface area of the insert (0.33 cm^2^).

### Whole Cell Electrophysiology

Whole cell patch-clamp recordings in the current-clamp mode were performed in clonal BEND-3 cells plated onto glass coverslips. Data were acquired at room temperature (20°C–22°C) using a commercially available amplifier (Axopatch 200B, Molecular Devices) and a Digidata 1440 A acquisition system (Molecular Devices). The pCLAMP software (version 10.0.2) was used for data acquisition and analysis. Patch pipettes were pulled from borosilicate glass capillaries on a Sutter Instruments P-1000 puller and had resistances of 3–5 MΩ. The extracellular solution contained (in millimolar): 138 NaCl, 2 CaCl_2_, 5.4 KCl, 1 MgCl_2_, 10 glucose, and 10 HEPES, pH 7.4 with NaOH. The pipette (intracellular) solution contained (in millimolar): 140 KCl, 2 MgCl_2_, 10 EGTA, 10 HEPES, 5 Mg-ATP, pH 7.3–7.4 with KOH. Resting membrane potential was determined after achieving the whole cell configuration; recordings were sampled at 10 kHz and filtered at 5 kHz via a four-pole Bessel low-pass filter.

### Permeability Assay

For permeability assay, inserts were moved to a new 24-well plate, and the receiving (lower) chambers were filled with 0.5 mL DMEM. Inserts (upper chamber) were washed with PBS and incubated at 37°C with 0.3 mL DMEM containing 1 mg/mL fluorescein isothiocyanate (FITC) dextran. Aliquots (50 µL) of medium in the lower chamber were collected at 30, 60, 90, and 120 min after the incubation; fluorescence was measured using a microplate reader (FLUOstar Optima, BMG Labtech, Ortenberg, Germany). Fluorescence values were normalized to that of a standard solution (DMEM with 1 mg/mL FITC-dextran); linear regression and line slopes were calculated after normalization to the fluorescence value of the first time point (30 min).

### Western Blot

Protein lysates were loaded on 4%–15% Mini-PROTEAN TGX precast protein gels at RT and transferred on PVDF membranes (Biorad, Milan, Italy). Membranes were incubated overnight with primary antibodies at 4°C; secondary antibodies conjugated to the fluorescent-dyes StarBright Blue 520 or StarBright Blue 700 were incubated for 1 h at RT. Stain-free gel and blot images as well as fluorescence signals were acquired with ChemiDoc touch imaging system. Images were analyzed by using Image Lab Software (version 6.1; Biorad, Milan, Italy). Intensities of the bands were normalized to total protein content in the lane, visualized through the stain-free blot imaging.

### RNA Extraction, Reverse Transcription, and PCR

Total RNA was extracted from BEND-3 cells using Norgen total RNA purification kit (Norgen Biotek, Thorold, Canada), according to the manufacturer’s instruction, and reverse transcribed to cDNA using sensiFast cDNA synthesis kit (Meridian Bioscience, Memphis). Quantitative-PCR experiments were run in a 7500 Fast Real-Time PCR system (Thermo Fisher, Monza, Italy) using sensiFAST SYBR-Green detection kit (Meridian Bioscience, Memphis). End-point PCR experiments were run using Wonder Taq Hot Start kit (Euroclone, Pero, Italy). Primers used in PCR experiments are listed in Supplemental Table S2.

### Water-Soluble Tetrazole Assay

Water-soluble tetrazole (WST) assay was performed using cell counting kit-8 (Dojindo EU GmbH, Munich, Germany), according to the manufacturer’s instructions. BEND-3 cells were seeded in 96-well plates (2,000 cells/well); WST solution (10 µL) was added to each well, containing 100 µL growing medium, and incubated at 37°C for 1 h. Absorbance at 450 nm was read using a microplate reader (FLUOstar Optima, BMG Labtech, Ortenberg, Germany).

### Drugs and Treatments

Retigabine was purchased from Valeant Pharmaceuticals (Laval, Canada), XE-991 and kainic acid (KA) from Tocris-Bio techne (Milan, Italy). Retigabine and XE-991 were dissolved in dimethyl sulfoxide (DMSO) and used at the concentration of 10 µM, whereas KA was dissolved in aqueous solution and used at 100 µM. Different experimental protocols were used for the experiments with BEND-3 cells. For the experiments in the Transwell system depicted in Fig. 2 (dextran permeability, TEER, and immunofluorescence), retigabine and XE-991 were diluted in a low-serum medium and incubated for 72 h. For the experiments with KA (Fig. 6), after 3 days in low-serum medium, cells were exposed to the following treatments: *1*) 0.1% DMSO for 2 h; *2*) 1 h DMSO followed by 1 h with KA plus DMSO; *3*) 1 h retigabine followed by 1 h with KA plus retigabine; *4*) retigabine for 2 h; all drugs were diluted in low-serum medium, and treatments were performed at 37°C. At the end of the 2-h treatment, medium with drugs was removed and cells were incubated with fresh low-serum medium for further 24 h, when functional and immunofluorescence experiments were performed. For WST assay depicted in Supplemental Fig. S5, the same treatment groups and procedures were used, but all experiments were performed with a growing medium. For the experiments evaluating the effects of Kv7 modulators on the expression of TJ markers (Fig. 3) and electrophysiological experiments, BEND-3 grown in monoculture was exposed for 24 h to CM and then incubated with retigabine, diluted in CM, for further 72 h. For WST assay depicted in Fig. 3, drugs were dissolved in growing medium and incubated for 72 h. For TEER experiments with primary ECs, retigabine and XE-991 were diluted in full medium and incubated for 72 h.

### Statistical Analysis

All data are expressed as means ± SD. One-way ANOVA test followed by a Bonferroni, Dunnett’s or Tukey’s multiple comparisons test, and Student’s *t* test (paired or unpaired) were used to determine statistical significance between groups, according to the different experiments. The significance level for statistic tests was 0.05 (differences were considered statistically significant when *P* < 0.05). *P* values for relevant comparisons are indicated in the graphs.

## RESULTS

### Expression of Kv7 Channels in the Blood Brain Barrier

**Figure 1.**
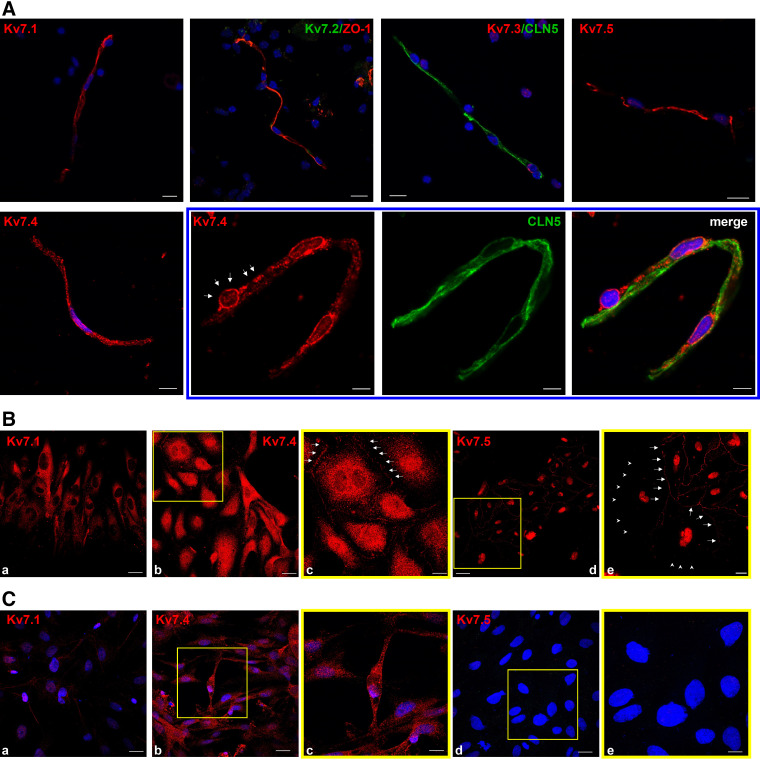
Expression of Kv7 channels in the BBB. *A*: immunofluorescence experiments showing the expression of Kv7 channels in BMVs, as indicated. Staining for ZO-1 and claudin-5, are displayed along with Kv7.2 and Kv7.3, respectively; nuclei (DAPI staining, blue) are also shown. Scale bar: 10 µm. Images within the blue frame depict Kv7.4 staining (red) in a pericyte (white arrows) surrounding a BMV; claudin-5 (green) and DAPI (blue) staining are also shown. Scale bar: 5 µm. *B*: immunofluorescence in primary ECs. Staining of Kv7 subunits is shown in red, as indicated. *c* and *e* (yellow frames) are enlargements of the region boxed in yellow in *b* and *d*, respectively. Arrows in *c* and *e* indicate possible plasma-membrane localization of Kv7.4 and Kv7.5; arrowheads in *e* show the absence of staining of Kv7.5 in regions where cell-to-cell contacts were not established. Scale bar: 20 µm in *a*, *b*, and *e*; 10 µm in *c*; 50 µm in *d*. *C*: immunofluorescence in primary PCs. Staining of Kv7 subunits are shown in red, as indicated. *c* and *e* (yellow frames) are enlargements of the region boxed in yellow in *b* and *d*, respectively. Nuclei (DAPI staining, blue) are also shown. Scale bar: 20 µm in *a*, *b*, and *d*; 10 µm in *c* and *e*. Images are representative of at least 30 microscopic fields each containing about 15 primary cells or 1–3 BMVs, acquired in at least six experimental sessions. BBB, blood-brain barrier; BMVs, brain microvessels; ECs, endothelial cells; PCs, pericytes; ZO-1, zonula occludens-1.

To investigate the possible expression of Kv7 channels in the BBB, we performed immunofluorescence experiments on isolated rat brain microvessels (BMVs). Centrifugation of brain samples on a dextran gradient led to the separation of 5–10 µm diameter capillaries, that expressed markers of TJs, such as Zonula Occludens 1 (ZO-1) and Claudin 5 (CLN5), as well as the endothelial marker CD31; BMVs tested negative for α-smooth muscle actin and β3-tubulin, two widely used markers of smooth muscle cells and neurons, respectively (Fig. 1*A* and Supplemental Fig. S1*A*). Immunofluorescence experiments using antibodies that specifically recognized the different Kv7 subunits (Supplemental Fig. S2) showed that BMVs expressed Kv7.1, Kv7.4, and Kv7.5 channels, whereas staining for Kv7.2 and Kv7.3 was negligible (Fig. 1*A*). Interestingly, expression of Kv7.4 was detected also in structures surrounding the small vessel, with the typical “bump-on-a-log” shape, that was not stained by the CLN5 antibody, corresponding to pericytes (Fig. 1*A*, *lower*, blue frame). Similar results were obtained with a second set of antibodies targeting each Kv7.1, Kv7.4, and Kv7.5 subunits (Supplemental Fig. S3). To confirm the expression pattern observed in BMVs, immunofluorescence experiments were performed in rat primary cultures of ECs and PCs. Expression of Kv7.1, Kv7.4, and Kv7.5 was detected in primary ECs, which showed the typical cobblestone-like shape, tested positive for the endothelial-specific proteins ZO-1, CLN5, and CD31 and negative for the pericyte-marker PDGFRβ (Platelet-derived Growth Factor Receptor β; Fig. 1*B* and Supplemental Fig. S1*B*). Staining for the three different Kv7 subunits was mostly localized intracellularly; however, Kv7.4 and Kv7.5, but not Kv7.1, could be also detected in the peripheral region of the cells, possibly suggesting a plasma-membrane expression (Fig. 1*B*, white arrows). Interestingly, expression of Kv7.5 could be observed at the cell-to-cell junction sites (Fig. 1*B*, *rightmost*, white arrows) but not in those regions where no contact with other cells was established (Fig. 1*B*, white arrowheads). In primary PCs, which expressed PDGFRβ and were negative for CD31 and CLN5 (Supplemental Fig. S1*C*), only Kv7.4 staining could be consistently detected; in contrast, Kv7.1 was weakly expressed, whereas Kv7.5 staining was negligible (Fig. 1*C*).

### Effects of Kv7 Modulators on Endothelial Permeability

**Figure 2.**
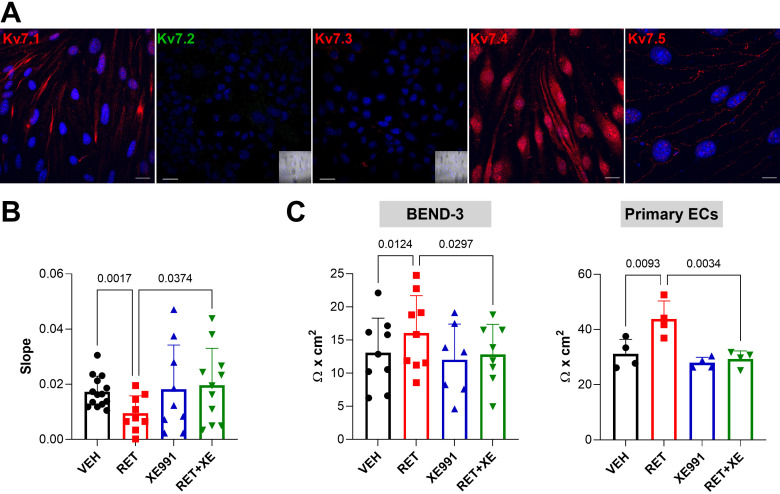
Effects of Kv7 modulators on BEND cells permeability. *A*: immunofluorescence images showing the expression of Kv7 channels in BEND-3 cells, as indicated. Nuclei (DAPI staining, blue) are also shown. *Insets* in Kv7.2 and Kv7.3 panels show brightfield images. Scale bar: 20 µm for Kv7.1, Kv7.2, Kv7.3, and Kv7.4; 10 µm for Kv7.5. Images are representative of at least 20 microscopic fields acquired in at least five experimental sessions. *B*: slope of normalized fluorescence signals from dextran-flux assay in BEND-3 cells calculated between 30 and 120 min, after exposure for 72 h to 0.1% DMSO (vehicle, black), 10 µM retigabine (red), 10 µM XE-991 (blue), and 10 µM retigabine plus 10 µM XE-991 (green). Data are expressed as means ± SD. *n* = 9–14; comparisons between experimental groups and relative *P* values are indicated in the graph (one-way ANOVA). *C*: scatter plot with bars showing transendothelial electrical resistance (TEER) measured in BEND-3 cells (*left*) and primary ECs (*right*) exposed for 72 h to 0.1% DMSO (vehicle, black), 10 µM retigabine (red), 10 µM XE-991 (blue), and 10 µM retigabine plus 10 µM XE-991 (green). Data are expressed as means ± SD. *n* = 7–9 (BEND-3); *n* = 4 preparations from 4 rats for primary ECs; comparisons between experimental groups and relative *P* values are indicated in the graph (one-way ANOVA). DMSO, dissolved in dimethyl sulfoxide; ECs, endothelial cells.

To ascertain whether Kv7 channels contributed to the regulation of BBB function, we took advantage of a widely used in vitro cellular model of BBB, namely BEND-3 cells cocultured with astrocyte-like cells (C6 glioma cells) in the Transwell system (32–34). Immunofluorescence experiments in BEND-3 showed an expression pattern of Kv7.1, Kv7.4, and Kv7.5 subunits similar to that observed in BMVs and primary ECs; expression of Kv7.2 and Kv7.3 was undetectable (Fig. 2*A*). In this experimental paradigm, we assessed the effects of specific pharmacological modulators of Kv7 channels on BBB permeability using FITC-conjugated dextran. Treatment for 72 h with the Kv7.2-5 opener retigabine (RET, 10 µM) reduced paracellular dextran flux by ∼50%. Incubation with the Kv7-specific blocker XE-991 (10 µM) alone did not modify dextran flux but prevented retigabine-induced decrease of membrane permeability (Fig. 2*B*). Further evaluation of BBB permeability was assessed in the same experimental paradigm by measuring trans-endothelial electrical resistance (TEER). Control BEND-3 cells showed a TEER of ∼15 Ω × cm^2^, a value consistent with previously reported studies (35, 36). Retigabine increased TEER by ∼20%, whereas XE-991, while failing to modify TEER when incubated alone, fully prevented the effect of retigabine (Fig. 2*C*, *left*). Strikingly, similar effects on TEER by Kv7 modulators were also observed in rat primary ECs (Fig. 2*C*, *right*).

We then assessed the effects of prolonged Kv7 activation on cell resting membrane potential (RMP), by means of current-clamp recordings. Treatment for 72 h with retigabine (10 µM) caused a hyperpolarization-shift of RMP by ∼10 mV when compared with control (vehicle: −15.53 ± 10.78 mV; RET: −25.34 ± 10.08; *n* = 8–13, *P* = 0.048).

Altogether, these results suggested that prolonged activation of Kv7 channels hyperpolarized ECs and reduced endothelial permeability.

### Molecular Mechanisms Involved in the Regulation of BBB Permeability by Kv7 Modulators

**Figure 3.**
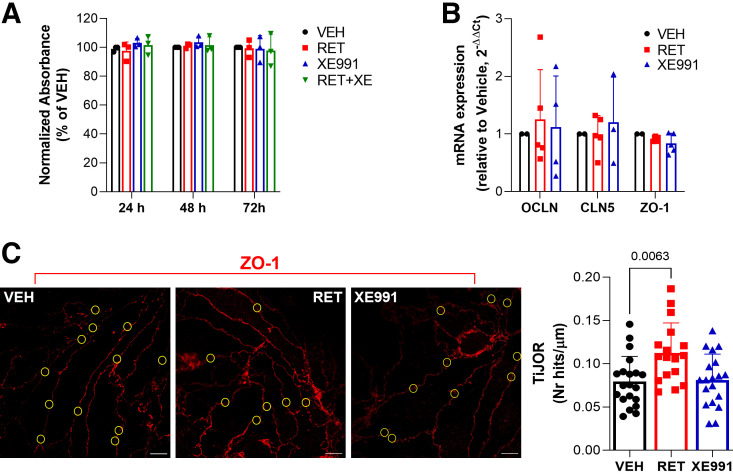
Effects of Kv7 modulators on BEND-3 cells proliferation, differentiation, and TJ integrity. *A*: scatter plot with bars showing water-soluble tetrazole (WST) assay in BEND-3 cells exposed for 24 h, 48 h, and 72 h to 0.1% DMSO (vehicle, black), 10 µM retigabine (red), 10 µM XE-991 (blue), and 10 µM retigabine plus 10 µM XE-991 (green). Data are expressed as percentage of the average of vehicle treated cells and shown as means ± SD. *n* = 3. *B*: quantitative PCR showing the expression of the transcripts encoding for occludin (OCLN), claudin-5 (CLN5), and zonula-occludens-1 (ZO-1) in BEND-3 cells exposed for 72 h to 0.1% DMSO (vehicle, black), 10 µM retigabine (red), 10 µM XE-991 (blue). Data are expressed as relative to vehicle-treated cells using the 2^−ΔΔCt^ formula. Data are shown as means ± SD. *n* = 3–5. *C*: *left*: representative immunofluorescence experiments showing ZO-1 staining in BEND-3 cells exposed for 72 h to 0.1% DMSO, 10 µM retigabine, and 10 µM XE-991. Yellow circles indicate gaps in ZO-1 staining along cell-to-cell junctions. Scale bar: 10 µm. *Right*: quantification of immunofluorescence images using tight junction organization rate (TiJOR). Data are expressed as means ± SD. *n* = 17–20 microscopic fields obtained in three experimental sessions. *P* value is indicated in the graph (one-way ANOVA). DMSO, dimethyl sulfoxide; TJ, tight junction.

Given that BBB permeability might be affected by ECs number and morphology, both parameters influenced by RMP and K^+^ channels activity (12, 16), we investigated whether pharmacological modulation of Kv7 channels altered cell proliferation and differentiation. Exposure of BEND-3 cells up to 72 h to retigabine (10 µM), XE-991 (10 µM), or retigabine plus XE-991 (both 10 µM) did not modify cell number (Fig. 3*A*). Moreover, expression levels for the TJs markers Occludin, CLN5, and ZO-1 did not change upon exposure to Kv7 modulators (Fig. 3*B* and Supplemental Fig. S4*A*). These results suggested that changes in cell proliferation or differentiation were not responsible for the reduced permeability observed upon Kv7 activation. However, further morphological analysis revealed that BBB integrity was strengthened by retigabine. Indeed, retigabine increased the tight junction organization rate (TiJOR), a validated method to assess BBB morphology (30), by reducing the number of occasional gaps in the staining of ZO-1 that could be observed along cell-to-cell contact regions both in coculture with C6 cells (Fig. 3*C*, yellow circles) and in monoculture in C6-conditioned media (Supplemental Fig. S4*B*); in contrast, XE-991 did not alter TiJOR (Fig. 3*C*).

### Expression and Function of Endothelial Kv7 Channels in Models of Kainic Acid-Induced BBB Damage

**Figure 4.**
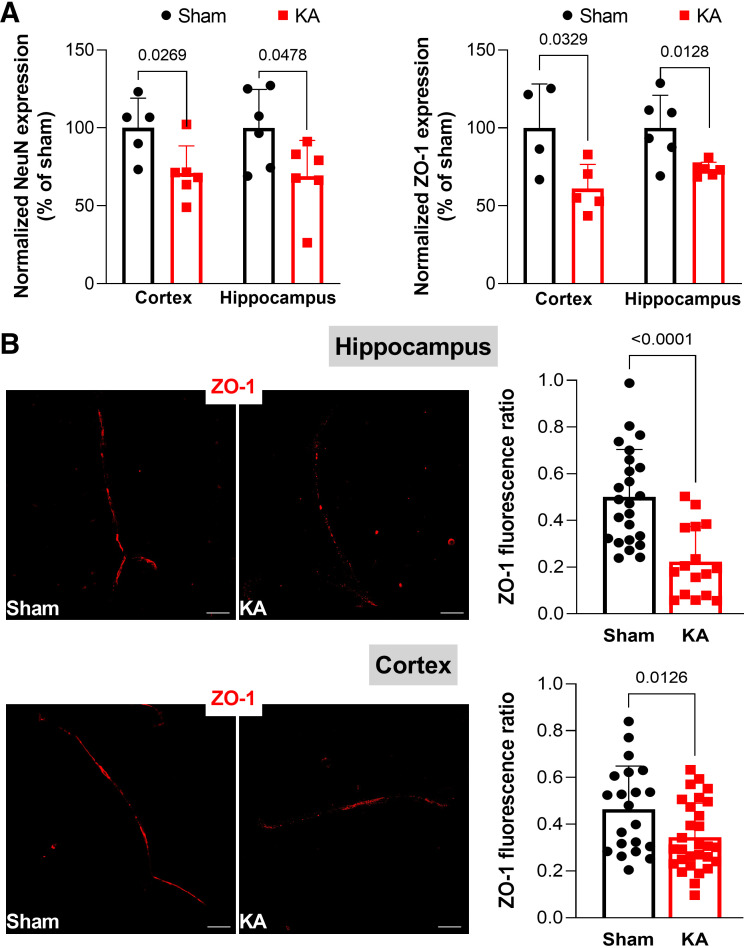
Expression of NeuN and ZO-1 in BMVs from KASE rats. *A*: Western blot showing the expression of NeuN (*left*) and ZO-1 (*right*) in homogenates obtained from cortex and hippocampus of sham- (black) and kainic acid-treated (red) rats. Data are expressed as percentage of the average of controls (sham) and shown as means ± SD. *n* = 5–6. *P* values are indicated in the graph (Student’s *t* test). *B*: representative immunofluorescence images showing ZO-1 staining in BMVs isolated from hippocampus (*upper*) and cortex (*lower*) of sham- and kainic acid-treated rats. Scale bar: 20 µm. Scatter plot with bars on the right show the quantification of ZO-1 staining in BMVs. Data are expressed as means ± SD. *n* = 16–28 BMVs obtained from 5 to 7 different animals per experimental point. *P* values are indicated in the graph (Student’s *t* test). BMVs, brain microvessels; KA, kainic acid; KASE, kainic acid-induced status epilepticus; ZO-1, zonula occludens-1.

Since BBB dysfunction is observed during epilepsy, we investigated whether Kv7 channels, here discovered to modulate ECs permeability, could participate in the pathological changes occurring at the BBB level during seizures (4). Western blot experiments performed in rat brain homogenates collected 24 h after kainic acid-induced status epilepticus (KASE) showed a reduction of the neuronal marker NeuN by ∼30% in both cortex and hippocampus (Fig. 4*A*, *left* graphs), suggestive of neuronal death, as previously reported (26). A decrease of ZO-1 expression in homogenates (Fig. 4*A*, *right* graphs), and a reduction of the continuity of ZO-1 staining in BMVs (Fig. 4*B*) were observed in both cortex and hippocampus from KASE rats, suggesting that TJs integrity was compromised.

**Figure 5.**
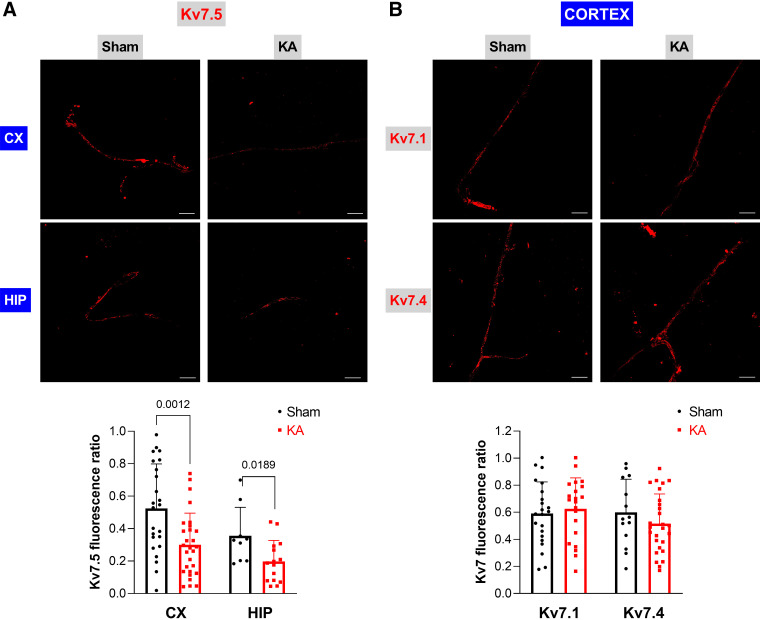
Expression of Kv7 channels in BMVs from KASE rats. *A*: representative immunofluorescence images showing Kv7.5 staining in BMVs isolated from cortex (*upper*) and hippocampus (*lower*) of sham- and kainic acid-treated rats. Scale bar: 20 µm. The graph shows the quantification of Kv7.5 staining in BMVs. Data are expressed as means ± SD. *n* = 24–29 BMVs obtained from 5 to 6 different animals per experimental point in the cortex; *n* = 9–15 BMVs obtained from five different animals per experimental point in the hippocampus. *P* values are indicated in the graph (Student’s *t* test). *B*: representative immunofluorescence images showing staining of Kv7.1 (*upper*) and Kv7.4 (*lower*) in cortical BMVs of sham- and kainic acid-treated rats. Scale bar: 20 µm. The graph shows the quantification of Kv7 subunits staining in BMVs. Data are expressed as means ± SD. *n* = 14–22 BMVs obtained from five different animals per experimental point. BMVs, brain microvessels; CX, cortex; HPI, hippocampus; KA, kainic acid; KASE, kainic acid-induced status epilepticus.

Interestingly, immunofluorescence experiments in the same preparations showed that Kv7.5 coverage of BMVs was reduced in KASE rats by ∼40% and 35% in cortex and hippocampus, respectively (Fig. 5*A*). In contrast, coverage of Kv7.1 and Kv7.4 in cortical BMVs was not changed in KA-treated rats (Fig. 5*B*). These results revealed a relevant downregulation of Kv7 channel expression after SE, selectively affecting Kv7.5 subunits.

**Figure 6.**
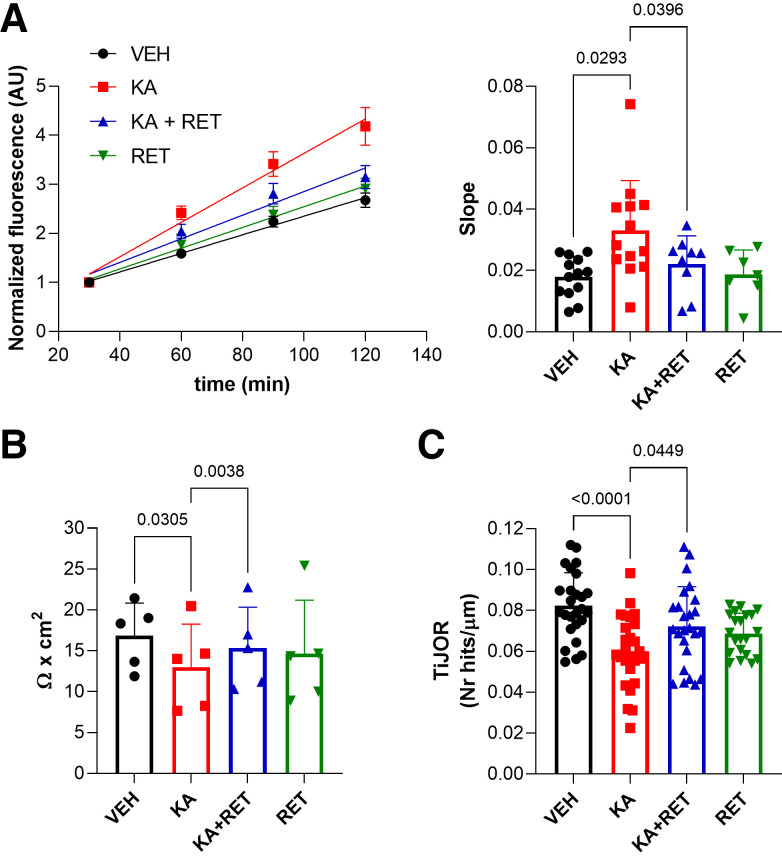
Effects of KA on BEND-3 cell permeability and integrity. *A*, *left*: dextran-flux assay in BEND-3 cells showing the normalized fluorescence over 2 h after treatment with 0.1% DMSO (vehicle, black line), 100 µM kainic acid (red), 100 µM kainic acid plus 10 µM retigabine (blue), and 10 µM retigabine (green); see materials and methods for further details. *Right*: slope of fluorescence signals calculated between 30 and 120 min. Data are expressed as means ± SD. *n* = 7–13; comparisons between experimental groups and relative *P* values are indicated in the graph (one-way ANOVA). *B*: scatter plot with bars showing transendothelial electrical resistance (TEER) measured in BEND-3 cells after treatment with 0.1% DMSO (vehicle, black), 100 µM kainic acid (red), 100 µM kainic acid plus 10 µM retigabine (blue), and 10 µM retigabine (green). Data are expressed as means ± SD. *n* = 5; comparisons between experimental groups and relative *P* values are indicated in the graph (one-way ANOVA). *C*: scatter plot with bars showing the tight junction organization rate (TiJOR) obtained by analyzing ZO-1 staining in BEND-3 cells after treatment with 0.1% DMSO (vehicle, black), 100 µM kainic acid (red), 100 µM kainic acid plus 10 µM retigabine (blue), and 10 µM retigabine (green). Data are expressed as means ± SD. *n* = 21–26 microscopic fields obtained in three experimental sessions; comparisons between experimental groups and relative *P* values are indicated in the graph (one-way ANOVA). DMSO, dimethyl sulfoxide; KA, kainic acid; RET, retigabine; VEH, vehicle; ZO-1, zonula occludens-1.

To further investigate whether the alteration of Kv7.5 channels had a pathological role in the dysfunction of the BBB, we evaluated in BEND-3 cells if the activation of Kv7 channels could prevent the endothelial damage caused by KA. BEND-3 cells express different classes of glutamate receptors including the KA receptors subtypes GLUK1 and GLUK5 (33), as also demonstrated by PCR experiments (Supplemental Fig. S5*A*). Incubation of BEND-3 cells with KA (100 µM) for 1 h did not affect cell viability (Supplemental Fig. S5*B*) but increased FITC-dextran flux by ∼50% (Fig. 6*A*), decreased TEER by ∼30% (Fig. 6*B*), and reduced TiJOR by ∼25% (Fig. 6*C*), suggesting the occurrence of the alteration of endothelial layer integrity. Interestingly, pretreatment with retigabine (10 µM) did not alter dextran flux, TEER, and TiJOR in absence of the insult, but prevented KA-induced effects (Fig. 6). Altogether, these results suggested that activation of Kv7 channels might prevent the pathological alterations of the BBB prompted by kainic acid.

## DISCUSSION

### Kv7 Channels as Novel Regulators of BBB Permeability

The BBB has gained increasing attention as a crucial regulator of CNS function both in physiological and pathological conditions. Among the players which have been shown to modulate BBB function, ion channels have emerged as important regulators of several processes including neurovascular coupling and barrier permeability (1).

In the present work, we provide evidence for the expression of three members of the Kv7 channels family in brain ECs and pericytes; moreover, we show that the pharmacological activation of endothelial Kv7 channels reduces BBB permeability by tightening cell-to-cell junctions and prevents kainic acid-induced damage.

In particular, our results revealed that Kv7.4 and Kv7.5 were abundantly expressed in brain ECs, with a distinct localization in the plasma membrane; Kv7.1 staining, with a predominant intracellular localization, could also be detected. These results are consistent with those from a recent single-cell RNA-sequencing study, where transcripts encoding for these Kv7 subunits have been detected in some subsets of brain ECs (37). Notably, the expression pattern of Kv7 subunits is similar to that observed in ECs from peripheral vessels (23, 24); in these cells, pharmacological evidence suggest that heteromeric channels formed by Kv7.4 and Kv7.5 play a more relevant functional role in the regulation of arterial contractility with respect to Kv7.1 subunit.

Interestingly, our experiments also revealed a strong expression of Kv7.4 in brain pericytes, whereas Kv7.1 and Kv7.5 were weakly expressed or undetectable, respectively. These results are consistent with the RNA expression profile identified in a previous study, with abundant expression of Kv7.4 and very low mRNA levels for Kv7.5 (37). In analogy to K_ATP_ subtypes linking neuronal metabolism, glucose availability, and capillary blood flow (38), pericyte Kv7 channels might contribute to the regulation of neurovascular coupling (39).

The role of ion channels in modulating BBB permeability has been mainly investigated in pathological conditions. For instance, it has been shown that the hyperpolarization mediated by the activation of K_ir_ channels (12) promotes ECs proliferation during hypoxia, whereas opening of the two-pore K^+^ channel TREK-1 reduces the levels of TJs markers and alters the expression of ECs adhesion proteins in a model of multiple sclerosis (15, 16). In the present study, we reveal that, in physiological conditions, opening of endothelial Kv7 channels with a selective activator (17) hyperpolarizes cell membrane, increases BBB integrity, and unveil a novel mechanism by which ECs function is regulated. Indeed, the decrease in ECs permeability induced by pharmacological activation of Kv7 channels was due to neither an enhanced expression of adhesion proteins nor an increase of cell proliferation; instead, activation of endothelial Kv7 channels augmented the sealing of TJs. Noteworthy, such tightening of ECs also occurred in the absence of astrocytes, which are known to modulate BBB properties and could have been potentially affected by drugs added in the Transwell upper chamber. Therefore, our data clearly indicate that endothelial Kv7 channels, by modulating membrane potential, play a relevant part in retigabine-induced decrease of BBB permeability. In epithelial cells, membrane depolarization triggers the phosphorylation of myosin light chain, thus promoting its interaction with actin. The subsequent contraction of the cytoskeleton can pull apart the cell-to-cell junction, thus causing the disruption of the BBB, and increasing its permeability (40). Moreover, depolarization can induce the phosphorylation of ZO-1, which leads to the redistribution of the protein away from the TJs with a consequent reduction of barrier integrity (41). Our data suggest that similar to epithelial cells, prolonged treatment with retigabine, promoting hyperpolarization of ECs, might oppose the activation of such pathways, thus favoring the sealing and the maintenance of the BBB. Interestingly, our immunofluorescence experiments showed a peculiar distribution of Kv7.5 subunits in the cell-to-cell contact regions; in these areas, transmembrane plaques formed by claudins and other adhesion molecules are connected to the cytoskeleton by adaptor proteins such as ZO-1 (42). The characteristic subcellular distribution of Kv7.5 subunits highlighted in the present work suggests the intriguing possibility of a privileged functional cross talk between ZO-1, whose distribution is modified by retigabine, and Kv7.5, and highlights a possible novel mechanism by which Kv7 channels may regulate BBB function.

### Selective downregulation of Endothelial Kv7.5 Channels in KASE

Several studies have highlighted that disruption of the BBB contributes to the onset and the progression of seizures (4, 43). Interestingly, a recent work showed that targeted knock-down of claudin-5 in mice led to spontaneous recurrent seizures, severe neuroinflammation, and increased mortality (44). At the same time, reduction of the expression of TJs markers such as ZO-1 and leakage of serum proteins into the cerebral parenchyma have been found in the hippocampi of patients with pharmaco-resistant temporal lobe epilepsy (TLE; 31); moreover, sustained epileptic activity is known to cause cytokines and glutamate release, which contribute to BBB damage (4). Overall, these findings suggest a strict and bidirectional influence of seizures and BBB dysfunction. In the present work, we demonstrated a possible pathological role for Kv7.5 channels in the structural and functional alteration of the BBB observed in the epileptic brain. Our immunofluorescence experiments showed a considerable and selective reduction of Kv7.5 expression in BMVs isolated from rats undergoing SE after administration of kainic acid, a widely used model of TLE. The morphological and functional rearrangement of the brain after the epileptic insult is a complex phenomenon that begins early after the onset of seizures and persists for a relatively long time (45, 46). Our results suggest that Kv7.5 might be an early marker/player of the pathological changes occurring in brain ECs after seizures, being altered already 24 h after the induction of SE. Moreover, our findings add new insight into the function of Kv7.5, whose pathophysiological role in the CNS has long been elusive. A role in epilepsy for Kv7.5 has been suggested by an early immunohistochemistry study in brain slices that showed a downregulation of Kv7.5 in hippocampal neurons from epileptic patients (47); more recently, mutations in Kv7.5 have been found in patients with intellectual disability, and epileptic encephalopathy showing clinical features that are quite different from those due to mutations in Kv7.2 and Kv7.3 (18, 48). Differences between Kv7.5 and Kv7.2/3 in terms of anatomical distribution within the CNS, ontogenesis, biophysical properties, as well as a specific subcellular neuronal localization have been proposed to explain the peculiar phenotype of patients carrying Kv7.5 variants (48). Irrespective of the suggested mechanism, such hypotheses have focused on the role of Kv7.5 at the neuronal level; our data showing that Kv7.5 is also expressed in brain capillaries and regulate BBB function offer an additional intriguing mechanism on how dysfunctional Kv7.5 channels might contribute to neurodevelopmental and/or epileptic phenotypes. To the best of our knowledge, these results are the first evidence of the alteration of the expression of an ion channel in the cerebral microvasculature following epileptic seizures.

### Pharmacological Activation of Kv7 Channels Prevents KA-Induced BBB Damage

One of the main consequences of increased neuronal activity associated to seizures is the massive release of glutamate, that, acting on different classes of receptors expressed in ECs, including the kainate subtypes, induces ECs apoptosis (49), increases BBB permeability (33), and alters the phosphorylation of TJs proteins such as occludin (32). To provide an in vitro model of glutamatergic-induced BBB dysfunction, we exposed BEND-3 cells to KA (100 µM). In our assays, retigabine prevented KA-induced increase of BEND-3 cells permeability and disruption of TJs, both phenomena indicative of BBB dysfunction. These results suggest a relevant role for endothelial Kv7 channels in the regulation of BBB during disease states. Alteration of BBB occurred in the absence of cell death, as instead reported by Barna et al. (33). Differences in the experimental protocol (exposure time) and in the model (primary vs. clonal ECs) might explain this discrepancy. Notably, in contrast with prolonged treatment, short exposure to retigabine did not affect BBB permeability. It is likely that opening of Kv7 channels, by opposing the acute and massive KA-mediated depolarization following the influx of Na^+^ and Ca^2+^ through ionotropic glutamate receptors (34, 49), might prevent the aberrant rearrangement of the cytoskeleton and the consequent BBB leakage.

The role of neuronal Kv7 channels in epilepsy is well known, and retigabine has been approved as an antiseizure drug in 2011. Our results showing that retigabine, in addition to direct effects on neuronal Kv7 channels, might exert an antiepileptic activity also through the action on Kv7 channels at the BBB level provide additional pathophysiological basis for the effectiveness of Kv7 channels activators in the therapy of epilepsy. Interestingly, retigabine has been shown to prevent cell death and BBB leakage in an in vivo model of brain ischemia; however, in this study, expression and function of Kv7 channels in the BBB have not been investigated (50).

### Study Limitations

Despite the novelty of the current findings, namely, that Kv7 channels are expressed in brain ECs and regulate BBB permeability in pathophysiological conditions, some limitations must be also highlighted. In fact, we were unable to provide electrophysiological evidence for an M-current in either primary or clonal ECs, despite several attempts with different patch-clamp configurations (i.e., whole cell and perforated patch), recording approaches and isolation protocols of ECs (data not shown). This is likely due to the small current size and its strict dependence on PIP2 (phosphatidylinositol 4,5-bisphosphate) abundance, which makes M current quite labile and prone to rundown in electrophysiological experiments. Notably, similar to our findings, the expression and functional role of Kv7 channels in peripheral ECs have been only assessed by immunofluorescence experiments and indirect functional readouts (23, 24). In addition, we did not study the specific contribution of endothelial Kv7 channels to BBB permeability in vivo. Since Kv7 channels are also abundantly expressed in neurons and their pharmacological modulation strongly influences neuronal function, it seems likely that BBB function, might be also indirectly affected by neuronal Kv7 channels. The use of animal models with cell type-specific Kv7 knockouts might discriminate the specific contribution of neuronal versus endothelial Kv7 channels on BBB permeability. Given the lack of Kv7 subunit-specific activators, a similar approach with subunit-specific Kv7 knockouts might corroborate our hypothesis of a preferential role for Kv7.5 (and possibly, Kv7.4) subunits in such process.

### Conclusions

Positioned at the interface between the bloodstream and neurons, the BBB is a unique structure that influences neuronal function; a bidirectional cross talk between neurons and BBB plays a key role in epilepsy. Therefore, defining the players that regulate BBB function and understanding the molecular mechanisms underlying the changes of permeability occurring in pathophysiological conditions are crucial to identify novel therapeutic targets. The data showed in the present work reveal Kv7 channels as new players in the regulation of BBB properties, allowing to speculate that their activation might be a possible strategy for the treatment of neurological diseases in which BBB dysfunction plays a major pathological role, such as traumatic brain injury and brain ischemia.

## DATA AVAILABILITY

Data are available upon request to the authors.

## SUPPLEMENTAL DATA

10.6084/m9.figshare.25036628 Supplemental Figs. S1–S5 and Tables S1 and S2: https://doi.org/10.6084/m9.figshare.25036628.

## GRANTS

This work was supported by the Italian Ministry for University and Research (MUR; PRIN 2020L45ZW4, granted to V.B.), the European Commission H2020 (UNICOM 875299, granted to M.T.), the European Joint Program on Rare Diseases (EJP RD) 2020 (TreatKCNQ, granted to M.T.). The project was also funded under the National Recovery and Resilience Plan (NRRP), Mission 4 Component 2 Investment 1.3, Call for tender No. 341 of 15/03/2022 of Italian Ministry of University and Research (MUR) funded by the European Union, NextGenerationEU [Project title ‘A multiscale integrated approach to the study of the nervous system in health and disease’ (MNESYS); code PE0000006, CUP D93C22000930002, MUR Concession Decree No. 1553 of 11/10/2022].

## DISCLOSURES

No conflicts of interest, financial or otherwise, are declared by the authors.

## AUTHOR CONTRIBUTIONS

M.T. and V.B. conceived and designed research; C.C., L.C., F.M., G.C., B.C., G.B., S.I., R.V., and V.B. performed experiments; C.C., L.C., F.M., G.C., B.C., G.B., S.I., R.V., and V.B. analyzed data; C.C., L.C., R.V., M.T., and V.B. interpreted results of experiments; C.C. and V.B. prepared figures; C.C. and V.B. drafted manuscript; V.B. and M.T. edited and revised manuscript; C.C., L.C., F.M., G.C., B.C., G.B., S.I., R.V., M.T., and V.B. approved final version of manuscript.
